# Understanding the Dimensions of Medical Crowdfunding: A Visual Analytics Approach

**DOI:** 10.2196/18813

**Published:** 2020-07-03

**Authors:** Jie Ren, Viju Raghupathi, Wullianallur Raghupathi

**Affiliations:** 1 Gabelli School of Business Fordham University New York, NY United States; 2 Koppelman School of Business Brooklyn College of the City University of New York Brooklyn, NY United States

**Keywords:** crowdfunding, medical crowdfunding, GoFundMe, fundraising, health care, health care affordability, patient, Facebook, fundraiser

## Abstract

**Background:**

Medical crowdfunding has emerged as a growing field for fundraising opportunities. Some environmental trends have driven the emergence of campaigns to raise funds for medical care. These trends include lack of medical insurance, economic backlash following the 2008 financial collapse, and shortcomings of health care regulations.

**Objective:**

Research regarding crowdfunding campaign use, reasons, and effects on the provision of medical care and individual relationships in health systems is limited. This study aimed to explore the nature and dimensions of the phenomenon of medical crowdfunding using a visual analytics approach and data crawled from the GoFundMe crowdfunding platform in 2019. We aimed to explore and identify the factors that contribute to a successful campaign.

**Methods:**

This data-driven study used a visual analytics approach. It focused on descriptive analytics to obtain a panoramic insight into medical projects funded through the GoFundMe crowdfunding platform.

**Results:**

This study highlighted the relevance of positioning the campaign for fundraising. In terms of motivating donors, it appears that people are typically more generous in contributing to campaigns for children rather than those for adults. The results emphasized the differing dynamics that a picture posted in the campaign brings to the potential for medical crowdfunding. In terms of donor’s motivation, the results show that a picture depicting the pediatric patient by himself or herself is the most effective. In addition, a picture depicting the current medical condition of the patient as severe is more effective than one depicting relative normalcy in the condition. This study also drew attention to the optimum length of the title. Finally, an interesting trend in the trajectory of donations is that the average amount of a donation decreases with an increase in the number of donors. This indicates that the first donors tend to be the most generous.

**Conclusions:**

This study examines the relationship between social media, the characteristics of a campaign, and the potential for fundraising. Its analysis of medical crowdfunding campaigns across the states offers a window into the status of the country’s health care affordability. This study shows the nurturing role that social media can play in the domain of medical crowdfunding. In addition, it discusses the drivers of a successful fundraising campaign with respect to the GoFundMe platform.

## Introduction

The crowdfunding phenomenon extends the traditional idea of fundraising into a contemporary internet platform–based funding vehicle [1-3]. In this exploratory study, we applied visual analytics to study medical crowdfunding, in which individuals raise funds for their medical treatment or for research projects via digital platforms such as GoFundMe [4-8].

Crowdfunding is an old phenomenon. Its earliest form, designed by Joseph Pulitzer, channeled Pulitzer’s newspaper to raise funds for the pedestal of the Statue of Liberty [1,9]. Similarly, this avenue has been used by artists, composers, and inventors to procure funding from backers willing to invest in their projects [10].

Crowdfunding has evolved into a powerful alternative to other traditional investment forms, such as venture capital, initial public offerings, and angel investments [1]. This trend has been facilitated by the ubiquity of digital media, including the internet, social media, e-commerce, and web-based advertising [11,12]. These platforms bestow comfort and ease in web-based transactions [1].

In the United States, the entrepreneurial culture has increased public awareness and the desire to support entrepreneurs [1,13]. As an alternative form of investment, crowdfunding offers opportunities for entrepreneurs to access various forms of funding [14]. As an example, Formlabs, a 3D printing startup, raised US $2 million in seed funding. The business used the Kickstarter crowdfunding platform to raise an additional US $2.9 million in the first year and US $19 million in the following year [1]. Kickstarter was initially used to raise small amounts of capital. However, its funding potential has progressively increased [15]. It is projected that the platform will generate more than US $300 million in funding by 2025 [16].

The process of crowdfunding begins with the entrepreneur’s pitch (or narrative) on details such as their background, funding-reward structure, and overview of the project or product for which funding is needed. This stage is intended for prospective backers [1]. The narrative is used throughout the funding period such that potential funders can evaluate the venture or the entrepreneur can make a funding decision. Owing to its potential, the phenomenon of crowdfunding is deployed in various domains, including law and medicine [6,17-20].

This study aimed to explore the nature and dimensions of the phenomenon of medical crowdfunding. It used a visual analytics approach and data crawled from the popular GoFundMe platform. This type of exploration sheds light on the characteristics and magnitude of the phenomenon, offering ethical, health, and social policy implications and recommendations [4-8]. This exploratory study addressed the following questions:

1. What are the dimensions of medical crowdfunding?
2. What factors create a successful medical crowdfunding campaign?

The next section provides the background information on crowdfunding, particularly medical crowdfunding. Following this is the outline of the methodology. Next, the results of visual analytics are presented, along with the scope and limitations of the study. Finally, the implications and future research are covered, along with the conclusions.

### Background

The success of crowdfunding in raising financial resources for new ventures has attracted the attention of entrepreneurs who explore this avenue for sourcing new ideas and projects [12,21-24]. In particular, crowdfunding facilitates entrepreneurs who are in the formative stages of gathering ﬁnancial resources to turn raw ideas into real business [21,23,25,26]. As this field grows, its conceptualization also evolves [12,15,21,23,27-30].

Research has identified key factors and definitions related to this phenomenon, including crowd, project creator (funding requester or fundraiser), and platform [21,27,28,30-35]. Some researchers suggest that a comprehensive deﬁnition of crowdfunding should include these elements as well as how each relates to or impacts others [21,34].

Microfinancing and crowdsourcing deﬁnitions have adopted a general conceptualization of crowdfunding [21,26,29,32,33,36-39]. The scope extends to both commercial and noncommercial forms of crowdfunding [14,21,27,35,40]. For example, Paschen [26] defined crowdfunding as “the outsourcing of an organizational function, through information technology, to a strategically deﬁned network of actors (ie, the crowd) in the form of an open call speciﬁcally, requesting monetary contributions toward a commercial or social business goal.” The objective of crowdfunding is that a crowd provides the “ﬁnancial resources either in form of donation or in exchange for some form of reward and/or voting rights to support initiatives for speciﬁc purposes” [41]. In the context of new ventures and entrepreneurial ﬁnancing, crowdfunding is defined as “the efforts by entrepreneurial individuals and groups—cultural, social, and for-proﬁt—to fund their ventures by drawing on relatively small contributions from a relatively large number of individuals using the internet, without standard ﬁnancial intermediaries” [29]. Other crowdfunding definitions, which focus on backers, are based on the contributions of an interested crowd of people in a specific phenomenon [42].

Using the context-speciﬁc approach, scholars have classified crowdfunding into several types [27,29,43-45]. Typically, 4 models can be broadly identified.

#### Donation

In this type of crowdfunding, funders support charity projects [46-49]. JustGiving, based in the United Kingdom, focuses on donation crowdfunding. Donors can be grouped based on the expectation of receiving rewards [26]. The first group (pure donors) ﬁnancially supports crowdfunding projects without either monetary or nonmonetary returns [26]. The second group does not receive monetary rewards. Instead, it receives nontangible rewards such as recognition or tokens [21,50].

#### Investment (Equity)

This crowdfunding option allows funders to invest in a project or business and acquire equity in return. SoMoLend in the United States and Crowdcube in the United Kingdom are examples of this model [46].

#### Lending (Debt)

This type of crowdfunding is when funders lend money to a project or business and expect repayment with or without interest. Kiva is a well-known example of lending.

#### Rewards

In this system, funders receive tangible or intangible items as rewards [46]. Fundraisers can select individual reward schemes for their contributions. This is a popular form of crowdfunding [14,27,35,40,44,46,51-55]. Examples include GoFundMe, Indiegogo, and Kickstarter.

Among platforms, GoFundMe, Indiegogo, and Kickstarter were the top crowdfunding websites in 2019 in terms of both fundraising and volume [56]. Other crowdfunding platforms include Fundly, JustGiving, Crowdrise, Indiegogo, Teespring, Patreon, YouCaring [50], Chuffed, ArtistShare, MightyCause, InKind, Crowdfunder, Kiva, and GiveWP. Fundraisers can find support for novel projects through crowdfunding, which would have been difficult through traditional funding sources. In addition, fundraisers have a choice in selecting platforms in which they do not have to pay commissions unless the effort is a success. For example, Kickstarter collects a 5% commission only if a project reaches its funding goal [50].

The number of crowdfunding platforms has grown in the past few years. Platforms such as Kickstarter, GoFundMe, and Indiegogo have shown increasing trends in fundraising [22]. According to AP News, the global crowdfunding market is expected to exceed US $28.77 billion by 2025 [57]. The potential for crowdfunding has urged regulatory efforts in the domain, such as the Jumpstart Our Business Startups Act [22]. In addition, many nonprofit businesses and governments are considering crowdfunding as a source for financing community programs aimed at serving the public [58]. In this regard, crowdfunding analytic tools can offer insights into the design of a successful fundraising campaign [22].

Next, this study focuses on the object of its research, that is, medical crowdfunding.

#### Medical Crowdfunding

In general, crowdfunding is being used for the development of community or social projects [6,29,58,59]. Health care can be considered both a community and social project because it incorporates programs such as poverty reduction and child education [6,29,59,60]. In this context, medical crowdfunding has emerged as a growing area of opportunity for crowdfunding [5,20,61-65].

What is the niche for medical crowdfunding? In recent years, crowdfunding has been adopted for entrepreneurial finance and litigation. More recently, individuals have deployed it for health issues and costs [6]. The increased need for funding health initiatives—the development of vaccines for public health or improving research protocols and systems—makes it a viable channel for deployment in the medical domain [5,6,66].

There are some renowned cases of medical crowdfunding [5]. For example, a campaign on RocketHub developed an affordable cost genome drug analysis test for patients [6,67]. This successful fundraising campaign spurred other campaigns like a rare genomic project for helping children with rare genetic diseases [68]. A campaign on Indiegogo analyzed microorganisms found on common public surfaces. The research aimed to prevent the spread of contagious diseases through mobile alerts [6,8]. These, and other similar projects, motivated contributions by creating donor awareness about the potential for medical research [6,69,70].

Some US-based websites are exclusively devoted to helping patients. These include GiveForward, GoFundMe, and YouCaring [6,20,61,62,66]. People who are ill or with disabilities resort to crowdfunding as a means to raise funds for meeting health care costs [4,20,61,66]. Environmental trends drive patients and caregivers to these platforms to raise funds for medical care. Factors include lack of medical insurance; aftereffects of the financial downturn in 2008; and lack of health care regulations, such as the Affordable Care Act [4,6-8,68]. Although crowdfunding can be used for many forms of medical needs, campaigns for acute and exceptional medical needs are more likely to be funded than those for chronic needs [63]. Medical crowdfunding campaigns can include raising funds for a variety of reasons, such as treatment of diseases in adults or children, hospital expenses, postoperative care, homecare needs, and general support in terms of drugs and postdiagnosis protocols, among others.

There are some dedicated medical crowdfunding websites. YouCaring had 15,880 active medical campaigns in 2016. FundRazr had 5326 campaigns [8]. GoFundMe reported an increase from 8000 campaigns (raising US $1.6 million) in 2011 to over 600,000 (raising nearly US $150 million) in 2014 [5]. GiveForward reported that medical-related needs are its most popular form of crowdfunding campaign [63]. These websites are expected to grow annually at a rate of 25% [71].

Despite this trend, little attention has been given to medical crowdfunding campaigns in terms of exploring reasons for use, impact on the provision of medical care, and individual relationships to health systems [4,8,17,66]. This exploratory study was conducted to address the paucity and obtain a panoramic view of the current state of medical crowdfunding. It explored the dimensions of medical crowdfunding as well as the factors of a successful campaign.

## Methods

### Visual Analytics Approach

This data-driven study used a visual analytics approach with primarily descriptive analytics [72,73] to obtain a panoramic insight into medical projects funded through GoFundMe. Visual analytics provides researchers and policy makers with effective ways to comprehend and analyze large datasets while acting on the findings in real time [74]. By integrating the computer’s capabilities with that of humans, visual analytics allows for the discovery of unexpected patterns and insights, which can lead to innovative and novel solutions [75,76]. It can address the challenge of information overload by translating information into viable opportunities, allowing researchers to examine results and the processes leading to those results [74,77,78]. The goal is to tell a compelling story through information visualization and the pillars of visualization, statistics, and data mining [75,77].

Descriptive analytics is based on the idea of describing data *as is* (without a preconceived assumption). Descriptive analytics is more data driven than other models; it allows for the understanding of past and current patterns and data trends, using the insight for informed decision making [72,79]. Through categorization, characterization, and aggregation or classification of data, information is presented visually in the form of meaningful charts and reports for analyzing business decisions [72,79].

In the current context of medical crowdfunding, we deployed visualization with descriptive analytics to address insightful questions such as the following. Drawing from the literature on medical crowdfunding and from general insights in the domain of crowdfunding, particularly in the context of web-based platforms, we explored the following in our study:

1. What factors lead to the success of a medical crowdfunding campaign?
2. Is there an optimum length for a fundraising campaign to be successful?
3. Do gender and age of fundraisers play a role in the amount of funds that can be raised?
4. Do pictures posted by fundraisers impact the success of the campaign?
5. What role does social media play in crowdfunding, particularly medical crowdfunding?
6. How does an analysis of medical crowdfunding campaigns across the states offer a window into the status of health care affordability in the country?

### The Platform: GoFundMe

The data source for this study is the GoFundMe crowdfunding platform, founded in 2010. It is the world’s largest crowdfunding platform in terms of the total funding amount raised and the total number of active campaigns [80]. GoFundMe campaigns include fundraising for several categories such as medical, memorials, emergency situations, and charitable causes.

In this study, we extracted data between June 19 and June 25, 2019, from the medical category of GoFundMe. We used the Selenium and Beautiful Soup packages in Python to crawl data from the medical category. Although it is possible that medical campaigns may exist in other categories, the medical category exclusively holds the bulk of these campaigns [7]. Therefore, we consider this to be an appropriate repository for medical crowdfunding campaigns. In addition, to ensure that only medical campaigns were included from this category in our data, we performed a manual perusal of the project descriptions upon extraction. Textbox 1 outlines the methodology used.

Table 1 describes the entities in the study and the variables for each entity. The entities in the crowdfunding study include the *fundraiser* (the person who is running the campaign and raising medical funds for himself or herself or another), the *campaign*, and the *donor*. We also show the source of each variable in terms of whether the code was directly downloaded from the data source or had to be coded after a manual perusal of the data. The variables are described in further detail in the context of the data analyses and results.

Using the web crawler, we collected data on 1000 medical crowdfunding campaigns. Three campaigns were eliminated for lack of data, resulting in a sample size of 997 fundraising campaigns. The raw data were extracted in a comma-separated value format and loaded into Tableau (Tableau Software Inc), a visual analytics tool. The following section discusses the results of the analyses.

**Methodology.**

**Data collection**

- GoFundMe

**Data preparation**

- Data crawled from GoFundMe, extracted in a comma-separated values format, and prepared for an analytic tool

**Platform selection and implementation**

- Tableau 2019.2

**Table 1:** Variables in the research.

Entity and variable		Description	Source of variable code
**Patient (for whom funds are being raised in the campaign)**			
	Gender	Gender refers to the gender of the patient for whom the campaign is developed (M: male, F: female)	Directly from the data source
	Age	Age refers to the age of the patient for whom the campaign is developed. People below the age of 18 years are considered children, while others are considered adults (C: children, A: adult)	Directly from the data source
**Campaign**			
	Group picture	Represents a picture that is posted in the campaign. If there is a single person in the picture, it is deemed to be a single picture. All other pictures are categorized as a group picture (G: group picture; S: single picture)	Manual review of data
	Status	Status provides details regarding the picture of the patient (P: picture in which the patient appears healthy and not in a medical setting; N: picture in which the patient appears ill and in a medical setting)	Manual review of data
	Position	Websites have multiple pages. Therefore, campaigns on the homepage are marked as *position 0*. Those on successive pages are marked with successive numbers (0-333)	Manual review of data
	Location	The geographical location of the fundraiser (city)	Directly from the data source
	Goal	The dollar amount that is sought through the campaign (US$)	Directly from the data source
	Length of fundraising	The time period for which the campaign has been active (months)	Directly from the data source
	Amount raised	Amount raised denotes the total dollar amount that the campaign generated (US$)	Directly from the data source
	Facebook shares	Number of people who shared the campaign link on their Facebook page (#)	Directly from the data source
	Favorite hearts	The number of people who liked the campaign on GoFundMe. This shows support toward the campaign (#)	Directly from the data source
**Donor**			
	Number of donors	Number of people who donated to a campaign (#)	Directly from the data source

## Results

### Distribution of Campaigns

**Figure 1:**
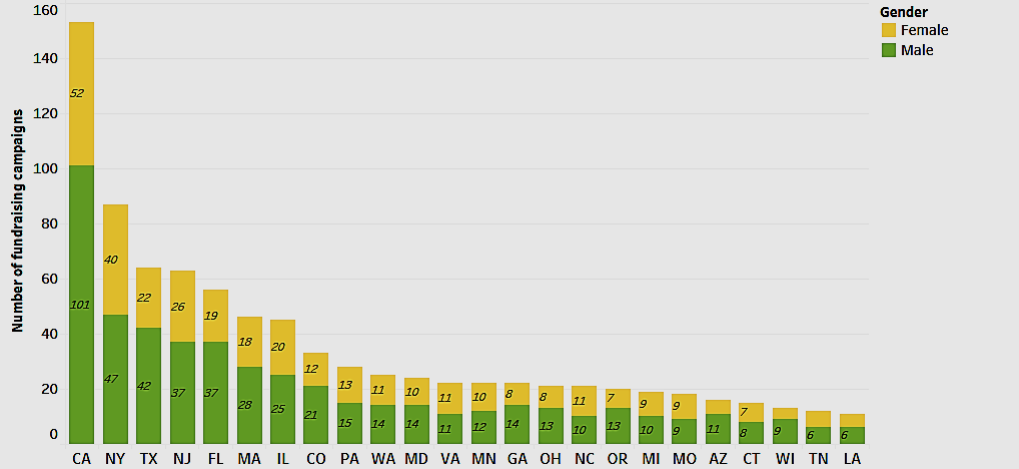
Distribution of fundraising campaigns by state.

We first explored the geographic distribution of the number of medical fundraising campaigns across various states in the United States (Figure 1). In addition to the geographical distribution, the figure depicts the distribution by gender.

Figure 1 shows that California has the highest number of fundraising campaigns (n=163), whereas the average number of campaigns for most states is approximately 20. This is followed by New York with 90, Texas with 65, New Jersey with 64, and Florida with 58 campaigns. We can only speculate on the distribution of fundraising amounts by state. For one, according to the 2019 US Census, these are also the states with a population of over 10 million [81]. It is also suggested that platforms such as GoFundMe, Fundly, and JustGiving are gaining popularity as people struggle to meet rising health care costs [82]. On the basis of this information, our results suggest the need for further empirical exploration on whether the high number of campaigns in these states is, in some way, a reflection of the high population that naturally entails a higher usage of the platform, or more people using the crowdfunding platform to cope with rising health care costs [81,82]. Figure 1 also reveals the analysis for gender. It shows that there are more campaigns by male patients 59.2% (59.2/100) compared with female patients 39.4% (39.4/100). In terms of age, our data show that most patients are primarily adults 80.9% (80.9/100).

### Factors Driving the Success of Campaigns in Terms of Amount Raised

**Figure 2:**
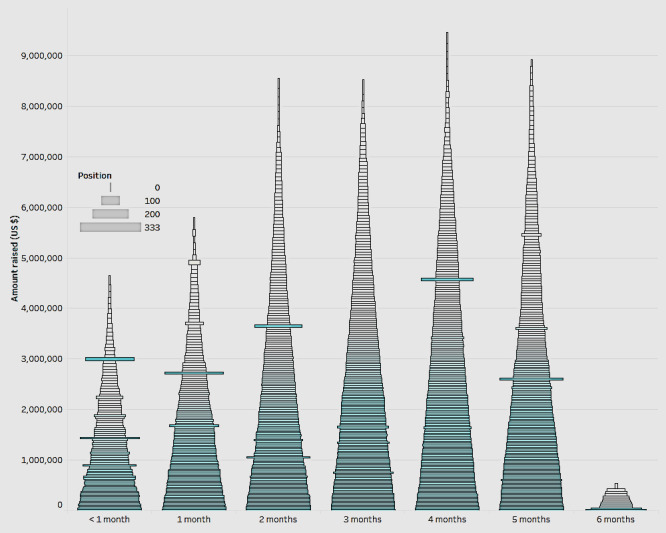
Impact of positioning and length of fundraising.

We then explored factors that can drive the success of a campaign. For one, if the position of the campaign posting on the website and the length of time that the fundraising campaign was active had any influence on the amount of funds raised (Figure 2). In Figure 2, the x-axis shows the duration of the campaign in months, and the y-axis shows the total amount raised. The position is represented by the size of the band for each campaign within each bar. The position of the campaign is the page on the website on which the campaign appears. The smallest bar represents a campaign positioned on the first page of the website (0). The size of the bar increases with the change in position on successive pages. The positioning of the campaign can play a major role in raising funds. Per the rationale, campaigns on the front pages receive higher visibility with donors. Thus, these campaigns have a higher chance of funding.

The results in Figure 2 show a positive correlation between the position of the campaign and the amount raised. Campaigns posted in the first few pages of the website do have a higher amount raised. With respect to the length of the campaigns, we found that campaigns that ran for 2 to 5 months received better funding and had a higher chance of meeting their goals. This shows that there is an optimal period for a campaign to remain active, beyond which the potential for increasing returns is questionable. Although this is an interesting finding, there is no prior research or literature to substantiate the reasons for the lack of an incremental effect on the amount raised after 5 months. We can only speculate on a few. For one, because of the increasing number of campaigns, after a period of time, donor attention may be diverted to other newer campaigns on the platform. Second, in medical crowdfunding, since the cause for funding is medical, this may have an effect on the perception of the sense of urgency of the need. A campaign that is open for a longer period may not convey an imminent sense of urgency as another that is open for a shorter term. However, further studies are needed to explore this in greater detail, incorporating more sophisticated techniques.

### Factors in Closing the Gap Between the Goal and the Actual Amount Raised

In the context of fundraising, it is important to identify which features of the campaign have a favorable influence in terms of closing the gap between the goal amount and the amount raised. The aim is to ensure that the difference is reduced and that the goal is reached.

**Figure 3:**
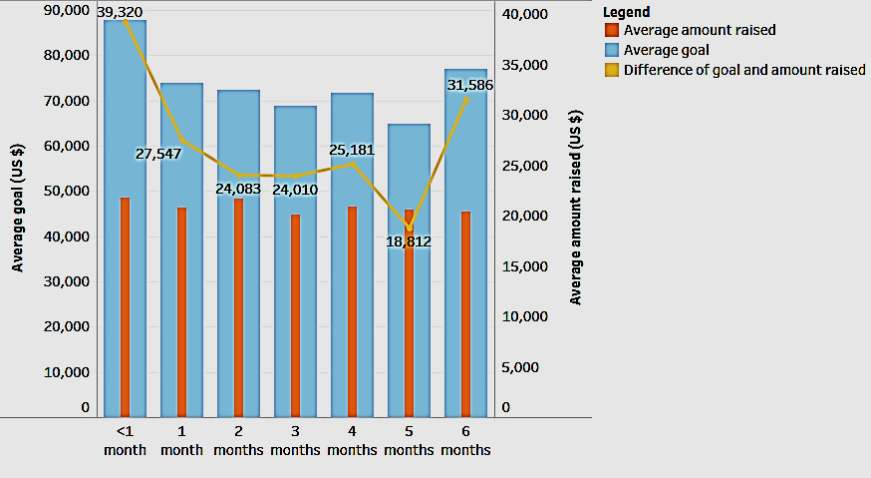
Role of the length of fundraising in closing the gap between the goal and the amount raised.

In this regard, we studied the role of the campaign length in influencing the gap between the goal and the amount raised. Figure 3 shows a combination line chart and a bar-in-bar chart. The blue bar shows the average goal of the fundraising campaign, the orange bar shows the average amount raised, and the yellow line chart shows the difference between the goal and the actual amount raised. These are depicted in the context of the length of fundraising (the x-axis).

According to Figure 3, as the length of the fundraising period (in months) increases, the difference between the goal and the actual amount raised decreases. The decrease lasts up to a period of 5 months. After that, the difference increases. This trend indicates that the optimal fundraising period to meet a goal is between 2 and 5 months.

**Figure 4:**
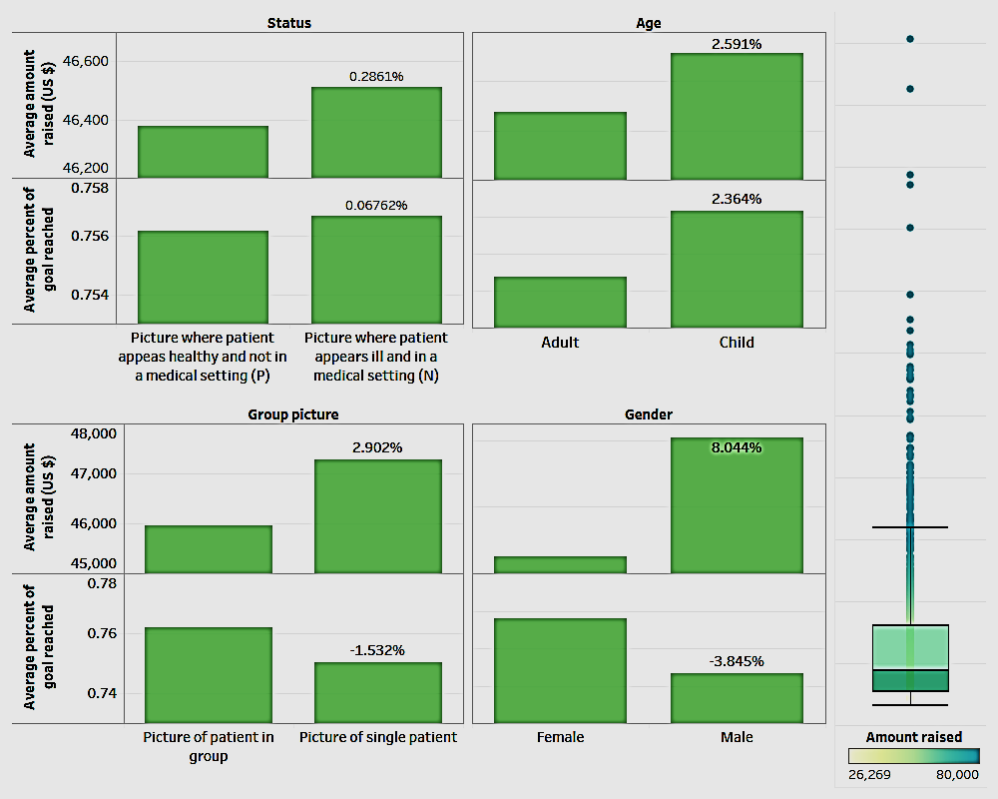
The average amount raised and percentage of goal reached by the different variables.

Next, we explored the role of other variables related to the patient (such as gender and age) and the picture (status and group picture) posted in the campaign (Figure 4). Gender refers to whether the patient is male (M) or female (F); age refers to whether the patient is a child (C) or an adult (A); status refers to the depiction of the status of the patient as reflected in the campaign picture*—*whether the patient appears healthy and not in a medical setting (P) or the patient appears ill and in a medical setting (N); and a group picture denotes whether it is a single picture (S) portraying only the patient or a group picture (G). Figure 4 uses a combination chart to show the average percentage of the goal reached and the average amount raised by each of the 4 variables.

In Figure 4, analysis by the status of the picture shows that campaigns with the picture of a patient appearing ill and in a medical setting (N) had a higher average amount raised and a higher average percentage of goal compared with campaigns with pictures where the patient appears healthy and not in a medical setting (P). On analyzing by gender, campaigns for male patients raised more funds (approximately 8%) on average than those for female patients; when comparing the average percentage of goal reached, campaigns for male groups lagged (by about 4%). When considering both the criteria of the average amount raised and the percentage of goal reached, campaigns for children raised more funds (about 2.5%) than those for adults.

**Figure 5:**
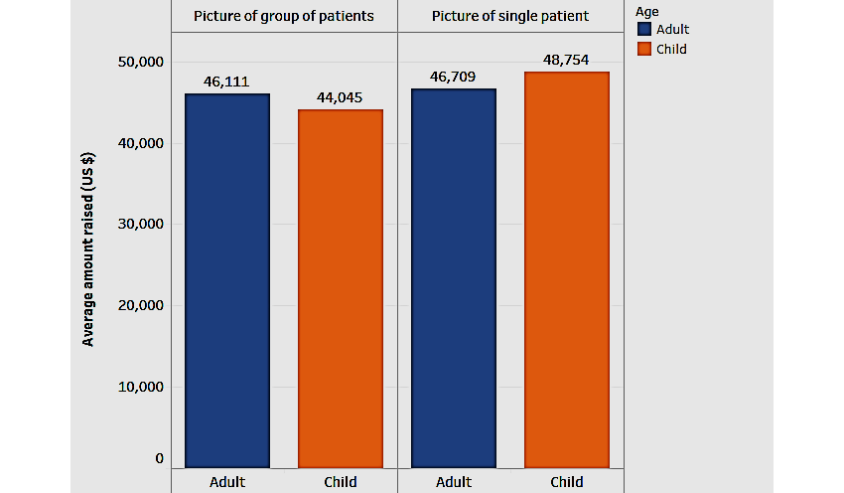
Influence of age and type of picture on the average amount raised.

While the above analysis revealed the separate influence of age and picture on the average amount raised, we now analyzed their combined influence on the average amount raised. Figure 5 depicts the amount raised for adult and pediatric patients, differentiated by the type of picture in the campaign (single: S or group: G).

Figure 5 depicts the amount raised for adult and pediatric patients differentiated by the type of picture in the campaign. Some campaigns use a picture showing a single patient while others show the patient in a group. Our findings from the figure show that for adult patients, the average amount raised with the picture of a single patient is slightly lower than that raised using a group picture. However, in the case of children, the trend is reversed, with a higher amount being raised with the picture of a single patient. Therefore, for adults, the difference is in the opposite direction and much less visible than for children. This holds interesting prospects for future research to further analyze the factors behind the differential effect being more pronounced for children. In addition, campaign developers can design campaigns with this differential in mind.

### Association of Popularity Measures of the Campaign With Amount Raised

**Figure 6:**
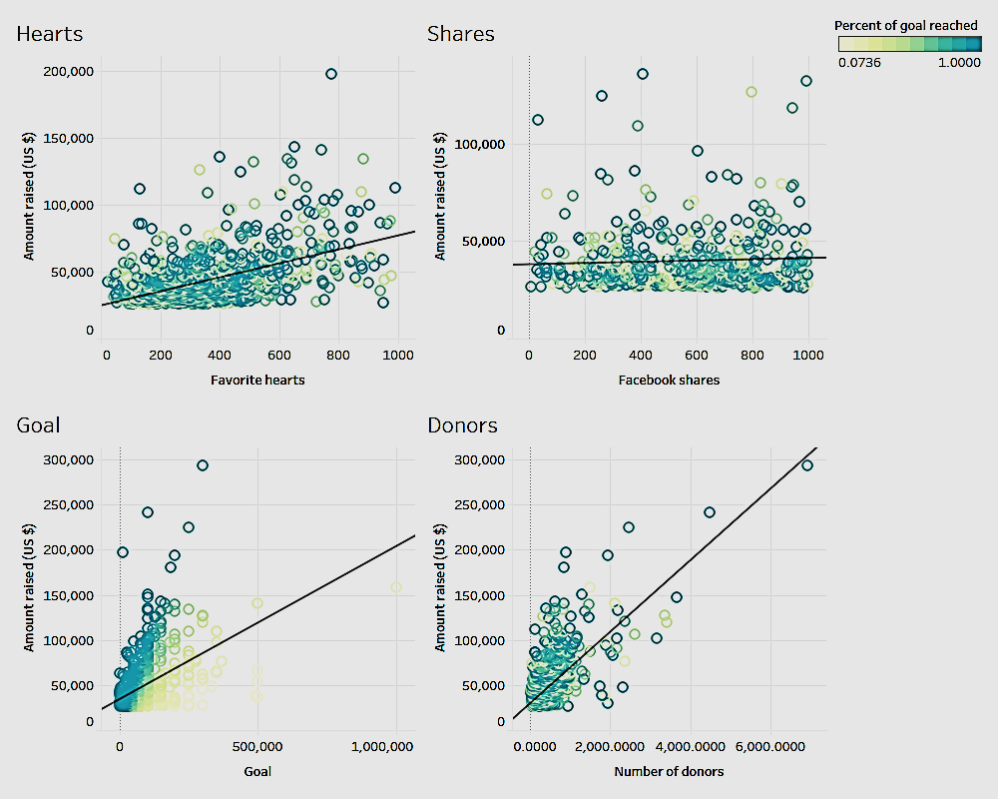
Association of amount raised with campaign popularity measures.

Campaigns have several measures that denote their popularity. In our study sample from GoFundMe, these include favorite hearts and Facebook shares. Favorite hearts denote the number of people who liked the campaign on GoFundMe. Facebook shares denote the number of people who shared the campaign on their social media platform, Facebook. In addition to these 2 measures, we also analyzed the influence of the goal and number of donors on the amount raised. We further showed the percentage of goals reached in the scatterplots (Figure 6). The dashboard in Figure 6 shows the associations for each of the 4 variables.

Figure 6 shows that favorite hearts have a significant and positive correlation (*P*<.001; *R*^2^=0.26) with the amount raised. Facebook shares did not have a significant correlation with the amount raised (*P*=.06). There was a significant positive correlation between funding goals and the amount raised for a campaign (*P*<.001; *R*^2^=0.30). Therefore, the higher the goal, the higher the amount raised. Finally, the number of donors had a significant positive correlation with the amount raised (*P*<.001; *R*^2^=0.47), which indicates that the higher the number of donors the higher is the amount raised. Keeping in mind that the average donation amount tends to be the same, the higher the number of donors correlates with the higher probability of raising a larger amount. From the analyses, we can see that in the association of goal with the amount raised, the colored bubbles show that the higher the goal amount, the higher the possibility of the percentage of the goal being achieved.

**Figure 7:**
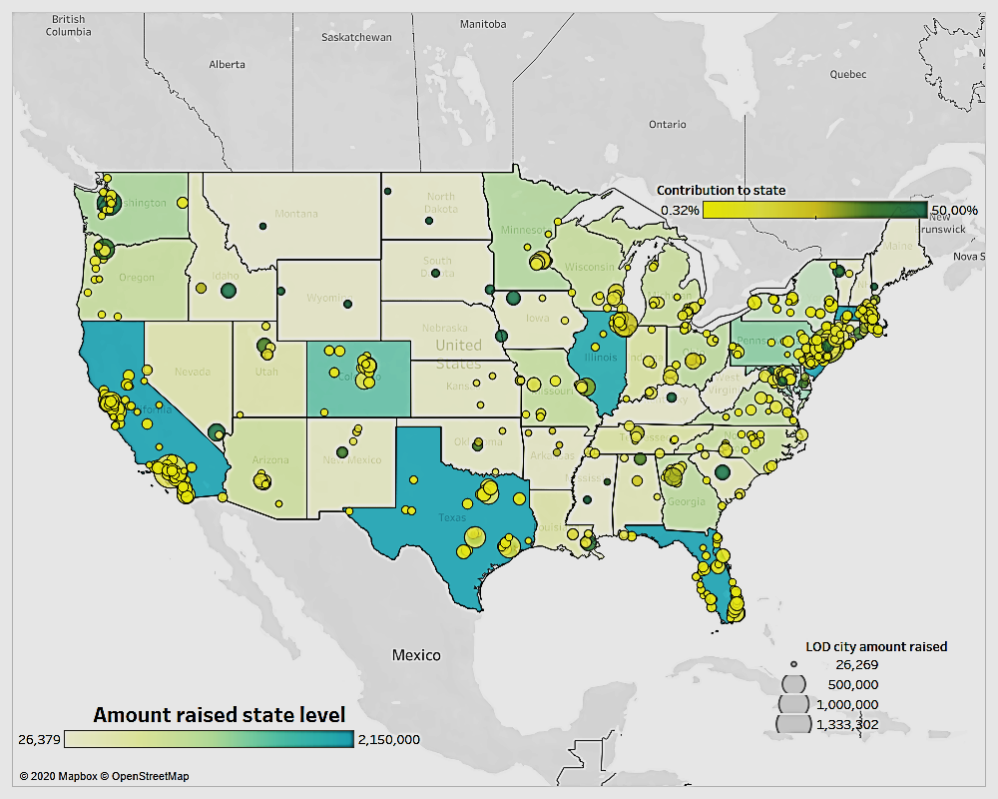
Amount raised by state and city.

Figure 7 illustrates the state- and city-level distribution of the amounts raised. This includes 3 characteristics: the intensity of color inside the states denotes the amount raised by the state, such that the darker colors show larger amounts raised; the bubbles inside each state represent the amount raised at the city level; and the bubble color indicates the contribution of the city-level amount raised to the state’s overall campaign amounts raised.

Figure 7 shows that California, New York, Texas, and Illinois have the highest amount raised compared with the other states. It also depicts clusters in the above states. These represent the locations of most of the crowdfunding campaigns. For example, areas around New York City and Los Angeles are the most concentrated in terms of campaigns created as well as amounts raised.

**Figure 8:**
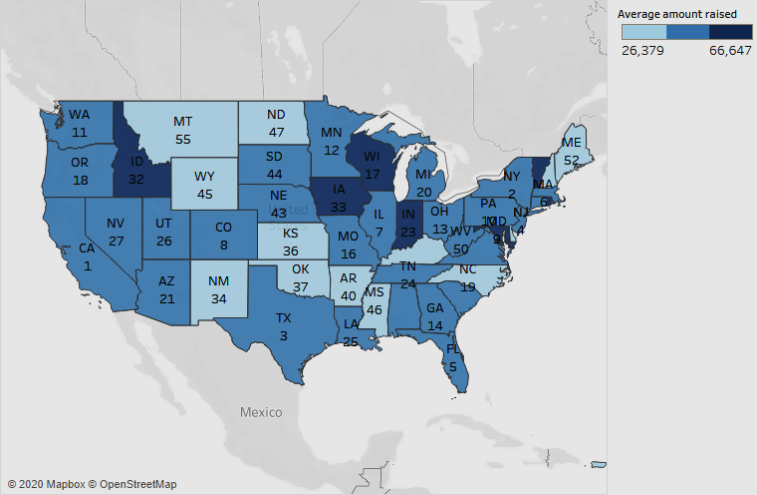
Average amount raised by state and ranking by total amount raised.

Along the lines of state-level distribution, we performed a state-level comparison of the average amount raised and the total amount raised (Figure 8).

In Figure 8, in addition to the average amount raised in each state (denoted by the color), the ranking in terms of the total amount raised is indicated as a label. The figure shows Iowa, Indiana, Wisconsin, and Idaho as the states with the highest average amount raised. However, in terms of the total amount raised, these states are ranked as 33rd, 23rd, 17th, and 32nd, respectively. In terms of the total amount raised, California ranks first, followed by New York, Texas, New Jersey, and Florida. These results highlight the potential for platforms such as GoFundMe to customize their campaign strategies based on the funding performance of the different states.

### Association of the Amount Raised With Relevant Campaign and Fundraiser Variables

**Figure 9:**
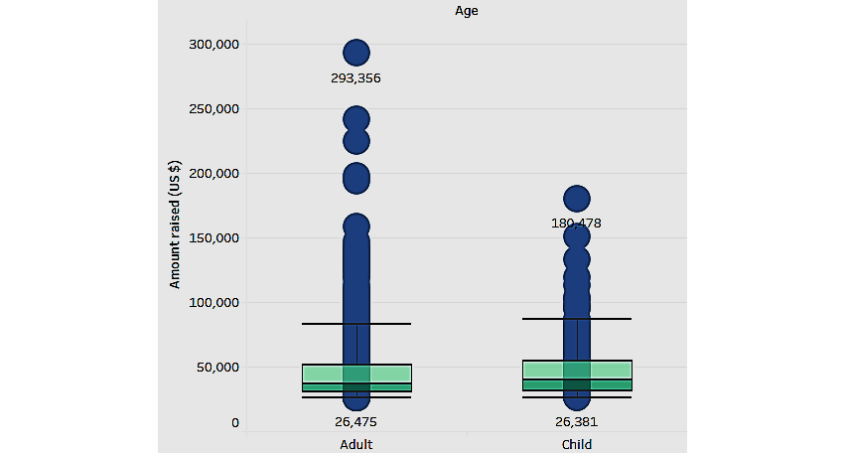
The median amount raised by age.

The box plot in Figure 9 shows the association of the amount raised with the age of the patients (child or adult) for whom the fundraising campaigns were developed.

Figure 9 shows that campaigns for adult and child patients have a similar median (approximately US $30,000 to US $40,000). However, the adult group has a higher biased distribution with more outliers. We propose that crowdfunding platforms pay special attention to these outliers because they may represent unique circumstances requiring specialized assistance and strategies for funding.

**Figure 10:**
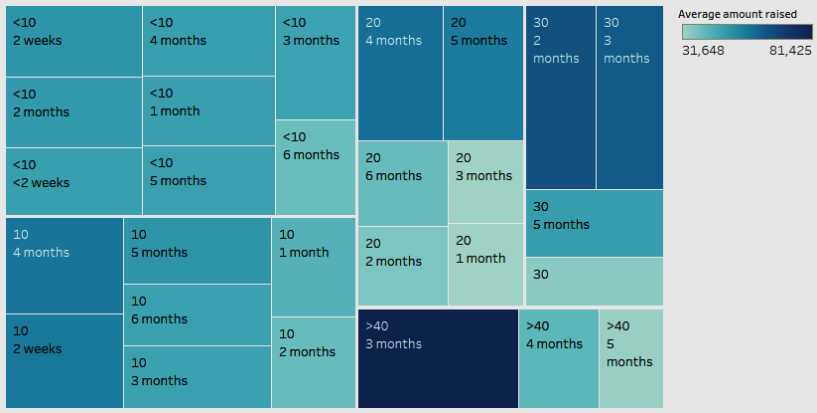
The average amount raised, number of updates, and length of campaigns.

We compared the amount raised with the number of updates and the length of the fundraising campaign (Figure 10). In the figure, the intensity of the color denotes the average amount raised, with the darker color representing higher amounts. The 2 labels inside the boxes represent the number of updates (top label) and the length of the fundraising campaign in months (bottom label). The updates represent those posted to the campaign regarding health and treatment.

Figure 10 shows that campaigns with an average of 30 to 40 updates can raise much higher amounts in a 2- to 3-month period. The highest average amount raised is for a fundraiser with more than 40 updates and a period of 3 months. It should be noted that these are average values. In calculating these averages, we did not consider the position or the goal of the campaign. They were assumed to be the same across campaigns. These results indicate that we can focus on the length and the number of updates as relevant criteria to achieve a fundraising goal.

### Trajectory of Donations and Influence of Favorite Hearts and Updates

**Figure 11:**
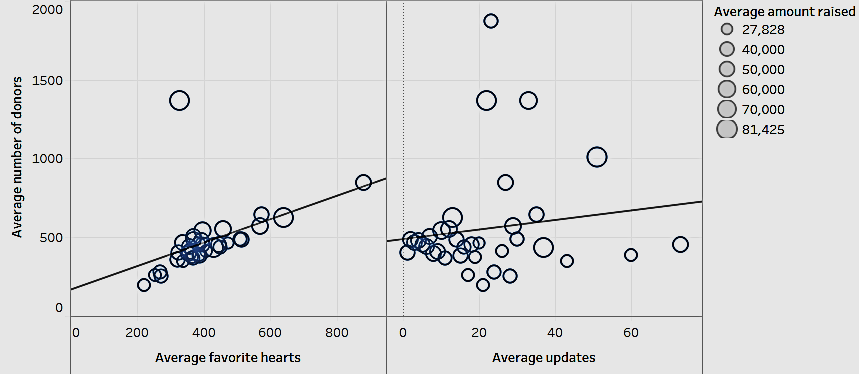
The average number of donors, favorite hearts, and updates.

Next, we looked for patterns in donations over time based on the number of updates or the number of favorite hearts in a campaign (Figure 11).

Figure 11 shows the relationship between the average number of donors with favorite hearts and the average number of updates in a campaign. The size of the bubbles in the scatterplots indicates the average amount raised. In Figure 11, we can see that the number of favorite hearts shows a significant positive association (*P*=.04) with the number of donors. Favorite hearts represent the number of people who liked the campaign on the GoFundMe website. It appears that people are more motivated to contribute if they see others endorsing a campaign in some manner. However, considering the lack of significance (*P*=.06) about the number of updates, it appears that donors focus on the initial story without following successive updates.

### Role of the Title and Description of the Campaign on Donations

**Figure 12:**
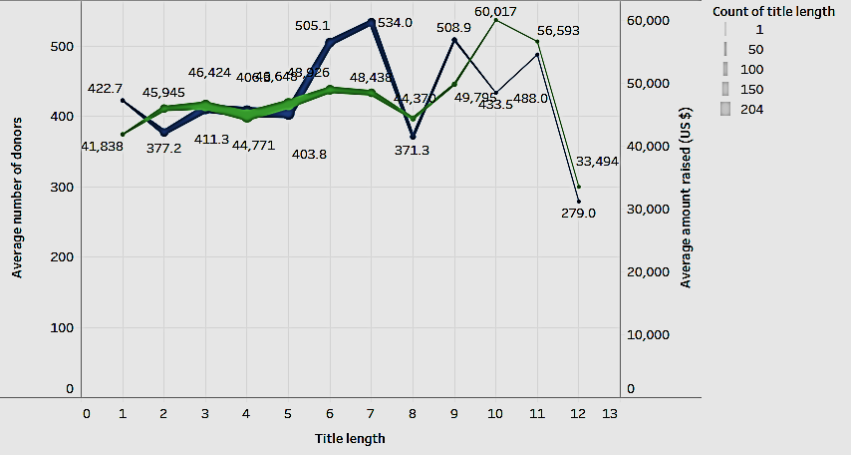
Influence of title length.

The title of the campaign was important. Therefore, we wanted to explore whether it influenced the average number of donors and the average amount raised (Figure 12). Figure 12 shows the two-line charts. The green line indicates the average amount raised, and the blue line indicates the number of donors.

Figure 12 shows that campaigns with titles having a length of 6, 7, and 9 words had a higher average number of donors. Titles with 10 or 11 words had a higher average amount raised. It can be inferred that the title plays an important role. An interesting title with 6 to 11 words can have a strong association with raising funds. The figure shows that most campaigns use 2- to 7-word titles. Future studies can explore the content of titles in addition to the length of in-depth associations.

Multimedia Appendix 1 depicts a word cloud for keywords that appear more than 500 times in the textual descriptions of campaigns.

A word cloud is a visual representation of the frequency and value of a word. It is used to highlight popular or trending terms based on the frequency of use and prominence in a corpus of text. The more times a keyword is present in a dataset, the bigger and bolder the keyword appears. Multimedia Appendix 1 shows that frequently occurring words include help, family, cancer, support, medical, treatment, time, and hospital. These represent the most discussed topics as well as the key factors in medical crowdfunding campaigns. The word cloud analysis shows that a large number of campaigns were designed to help with costs related to cancer treatment.

## Discussion

### Principal Findings

The visual analysis provides key insights that can help in developing successful medical crowdsourcing campaigns. States such as California and New York show the highest number of campaigns in the country. This indicates the need for further empirical investigation into a few possibilities: whether the high population in these states [81] naturally suggests higher platform usage or being that the objective of medical crowdfunding is primarily to meet medical costs [82], whether high platform usage actually reflects people reaching out for health care needs [81,82]. This study showed that some factors influence the amount raised in a medical crowdfunding campaign. For example, the positioning of a campaign is crucial. Posting a campaign within the first 10 pages of the website enhances its visibility to donors.

In terms of donor motivation, people are typically more generous in contributing to campaigns for children. It is also interesting to note the dynamics that a picture brings to the fundraising potential of a medical campaign. This study showed that a single patient picture is more effective for children. A group picture is more effective when fundraising for adults. A picture depicting the current medical condition of the patient (as severe) is more effective in motivating donations than one that depicts normalcy in the patient’s condition.

We draw attention to the importance and effectiveness of the title in a medical crowdfunding campaign. In this regard, an optimum length contains 6 to 11 words. Regarding the role of gender and age of patients for whom the campaigns are developed, it appears that campaigns for female children are more successful, raising up to 78% of the goal amount.

Finally, an interesting trend in the trajectory of donations is that the average donation decreases with an increase in the number of donors. This indicates that the first donors tend to be the most generous. These initial donations are typically substantive, suggesting that they may be from the family or friends of the fundraiser. The public at large seems to donate marginally less than the initial donors.

### Scope and Limitations

This study has some limitations. We crawled the data during a specific time period, that is, 2019. Therefore, it provides a snapshot of crowdfunding activity. Future studies should span a longitudinal timeframe and a more expansive set of variables. In addition, although we used a visual analytics method, alternative techniques such as data mining (ie, clustering, association) can be deployed. Although visual analytics, as a methodology, offers descriptive analysis, further empirical investigation is needed to draw quantitative conclusions. Furthermore, although the research focuses on the amount raised, goal, and position of the campaign, information on the fundraiser’s background is not included. Access to fundraiser information would help ascertain whether certain profiles are more successful in attracting donations. Including specific information about the donors may also enable researchers to conclude whether the donor knew the patient or how many people donated to an unknown cause. Future studies can analyze differences in the type of patients for whom funds are raised (eg, medical expenses for a patient or pet). In addition, future studies can incorporate details on the insurance coverage of fundraisers. This might help ascertain their actual medical requirements. It will also be interesting to determine the current situation of fundraisers with regard to whether the treatment is completed or ongoing.

### Implications

Despite these limitations, this research presents numerous contributions to the literature on medical crowdfunding and health care. This research highlights factors that are key to the success of a medical crowdfunding campaign. It also demonstrates the critical role of social media in the domain of health care. For example, research shows that campaigns with photos, frequent updates, and descriptions get more hearts (likes) and Facebook shares.

Through this research, we show how data-driven analytics can help donors make educated, fact-based contribution decisions in medical crowdfunding. The visual analytics methodology offers a holistic perspective on the phenomenon, including insight for policymaking in the arena of medical crowdfunding. Furthermore, this study offers insight into the geographic distribution of crowdfunding campaigns. This highlights the need for advanced analytics to empirically explore the contributing factors for differentials in platform use and fundraising success by state.

### Conclusions and Future Research

This study focused on the dimensions and factors of a medical crowdfunding campaign using the most popular platform, GoFundMe. We examined the relationship between the use of social media, the characteristics of a campaign, and the potential for fundraising. The analysis of medical crowdfunding campaigns across the states offers a window into the status of health care affordability in the United States. The research shows the nurturing role that social media can play in the domain of medical crowdfunding. We also add to the drivers of a successful fundraising campaign with respect to the GoFundMe platform.

This research is significant because the topic of medical crowdfunding has been gaining public attention. Ethical concerns have been raised in relation to these kinds of campaigns. Most of these concerns focus on issues relating to both fundraisers and donors, such as exposure to fraudulent campaigns, loss of privacy, and fairness in fund distribution [6,8,65]. There is also a debate on whether campaigns can be designed to raise funds for medical treatment or conditions related to pets.

In the meantime, systematic efforts to comprehend the scope of these ethical concerns are lacking. For example, a search on Google Scholar using the terms *medical* and *crowdfunding* returned only 3 results focusing on crowdfunding specifically for covering medical expenses rather than for research [6,8,68]. Researchers and academicians can add insight into the phenomenon of medical crowdfunding given the interest in the social dimensions of health care combined with the ability to deploy a variety of methods from different disciplines [6,8,65]. For instance, health geographers can offer insight into the spatial dimensions of campaigns, such as the extent of the donor network from an ethics of care approach [6,8,68]. Health economists can explore the regulatory aspects related to the possible competing interests of parties in medical crowdfunding. Health informatics experts can incorporate leading-edge technology such as text analytics or sentiment analysis to analyze whether the severity of the medical condition influences the funds raised for medical crowdfunding campaigns.

Drawing from our research and the related literature, we propose ethical and social considerations for future medical crowdfunding research. First, it is important to explore how medical crowdfunding impacts the national phenomenon of health care, specifically affordability and cost of care. The exploration of whether the age group of patients has a differential impact on fundraising potential is also important. Second, crowdfunding platforms are all technology based. Therefore, research needs to explore fundraiser information privacy and security [6,8,65]. Third, the associated aspect of regulating misuse of platforms for fraudulent campaigns, including misrepresenting the severity of illness for securing funds or misappropriating funds, needs to be investigated [8,62]. Fourth, the results of the study emphasize the role of social media endorsements for crowdfunding campaigns, such as Facebook shares. It also questions whether social media companies should regulate the authenticity of endorsements as well as their role in influencing potential donors. At this stage, more social, political, and economic issues will continue to be explored as the prevalence and popularity of crowdfunding technology platforms rise.

## Appendix

Multimedia Appendix 1 Word cloud of frequently used words in textual descriptions.
