# Supplementary figures and images for: Understanding the Dimensions of Medical Crowdfunding: A Visual Analytics Approach
Source: J Med Internet Res. 2020 Jul 3;22(7):e18813. doi: 10.2196/18813 (PMC7367538; doi:10.2196/18813)

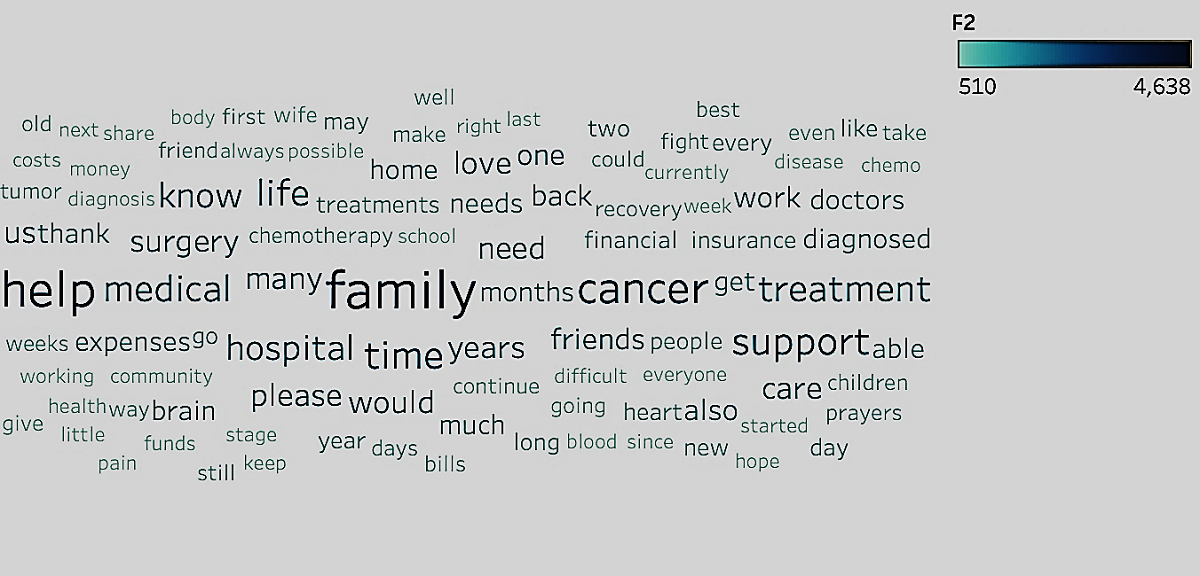

Supplement: Multimedia Appendix 1 [file jmir_v22i7e18813_app1.png]
